# Chinese Herbal Medicine for Functional Abdominal Pain Syndrome: From Clinical Findings to Basic Understandings

**DOI:** 10.1155/2016/8652523

**Published:** 2016-06-05

**Authors:** Tao Liu, Ning Wang, Li Zhang, Linda Zhong

**Affiliations:** ^1^Institute of Digestive Diseases, Longhua Hospital, Shanghai University of Traditional Chinese Medicine, Shanghai 200032, China; ^2^School of Chinese Medicine, Li Ka Shing Faculty of Medicine, University of Hong Kong, Pok Fu Lam, Hong Kong; ^3^School of Chinese Medicine, Hong Kong Chinese Medicine Study Centre, Hong Kong Baptist University, Kowloon Tong, Hong Kong

## Abstract

Functional abdominal pain syndrome (FAPS) is one of the less common functional gastrointestinal disorders. Conventional therapy has unsatisfactory response to it so people turn to Chinese medicine for help. Currently, we reviewed the whole picture of Chinese herbal medicine (CHM) clinical and basic application in the treatment of FAPS, especially the traditional Chinese medicine (TCM) syndrome, the single herb, and Chinese medicine formulae, thus to provide a solid base to further develop evidence-based study for this common gastrointestinal complaint in the future. We developed the search strategy and set the inclusion and exclusion criteria for article search. From the included articles, we totally retrieved 586 records according to our searching criteria, of which 16 were duplicate records and 291 were excluded for reasons of irrelevance. The full text of 279 articles was retrieved for detailed assessment, of which 123 were excluded for various reasons. The number one used single herb is Radix Ginseng. The most common syndrome was* liver qi depression*. The most frequently used classic formula was Si-Mo-Tang. This reflected the true situation of clinical practice of Chinese medicine practitioners and could be further systematically synthesized as key points of the therapeutic research for FAPS.

## 1. Introduction

Functional abdominal pain syndrome (FAPS) is one of the less common functional gastrointestinal disorders [1]. It also has less investigation compared to other functional digestive disorders, namely, irritable bowel disorder (IBS) and functional constipation (FC). The disorder is characterized by continuous, almost continuous, or at least frequently recurrent abdominal pain that is poorly related to bowel habits and often not well localized [2]. Until currently, the definition and diagnosis of FAPS have only been recognized. The Rome III classification has symptom-based criteria to diagnose FAPS [3]. Diagnostic criteria (criteria fulfilled for the last 3 months with symptom onset at least 6 months before diagnosis) for functional abdominal pain syndrome must include all of the following:

1. Continuous or nearly continuous abdominal pain.
2. No or only occasional relationship of pain with physiological events (e.g., eating, defecation, or menses).
3. Some loss of daily functioning.
4. The pain is not feigned (e.g., malingering).
5. Insufficient symptoms to meet criteria for another functional gastrointestinal disorder that would explain the pain.

Another feature about it is that FAPS appears highly related to alterations in endogenous pain modulation systems, which is consistent with dysfunction of descending pain modulation and cortical pain modulation circuits.

The epidemiology of FAPS is very limited because of the lack of available data and the difficulties in establishing a diagnosis that can be differentiated from other more common functional gastrointestinal disorders [4]. Some research indicated that the prevalence of FAPS ranges from 0.5% to 2% in North America and a female predominance was noted (F : M = 1 : 1.5). A substantial proportion of patients are referred to gastroenterology practices and medical centers; they have a disproportionate number of health care visits and often undergo numerous diagnostic procedures and treatments.

The clinical manifestation is characterized by the presence of continuous or frequently recurrent abdominal pain associated with loss of daily functioning [3]. FAPS is better understood as an abnormal perception of normal (regulatory) gut function, instead of a true motility disorder. Therefore, patients with FAPS will not typically experience relief of pain after defecation (a pattern that is characteristic for irritable bowel syndrome [IBS]), supporting the contention that disturbances in bowel motility do not fully explain the pain [1, 2, 4, 5]. In contrast to other functional gastrointestinal disorders, treatments for patients with conventional medicine are empirical and not based on results from well-designed clinical trials. It focuses on establishing an effective patient-physician relationship, following a general treatment approach, and offering more specific management that often encompasses a combination of treatment options [1–7]. So far, there is no satisfactory therapy for FAPS and many people seek Chinese medicine (CM) for help.

According to CM, FAPS usually belongs to the CM syndrome of “abdominal pain,” which related to exogenous evils, intemperate diet, yang deficiency, and liver qi stagnation. In summary, FAPS in CM could be classified into two main types. One is excessive syndrome, including exogenous evils, intemperate diet, dampness-heat, and qi stagnation; the other is deficient syndrome, including yang deficiency and qi deficiency. When exogenous cold and wind attack the abdomen or excessive raw and cold foods injure the stomach or abdomen, all these will impair and block qi movement. Such blockage will lead to abdominal pain. Dampness and heat will also invade and lodge qi movement [8].

The treatment of CM for FAPS is based on the criteria of syndrome differentiation [9]. In general, herbal treatment and acupuncture are the main two approaches. In this paper, we will focus on Chinese herbal medicine (CHM), especially the single herb, Chinese medicine formulae, CM syndromes, and their related Chinese medicine syndrome. The key clinical syndromes of FAPS include five main types, which are* blockage by cold, qi stagnation, dampness-heat, yang deficiency, *and* qi deficiency*. The first three types belong to excessive syndromes and the latter two types belong to deficient syndrome. When treating abdominal pain, be careful to identify the affected meridian, ascertain whether it is due to qi or to cold or heat, and differentiate between excessive and deficient syndrome. For abdominal pain of excessive syndrome emphasize expulsion of disease evil and relief of blockage [10–12]. For abdominal pain of deficient syndrome emphasize warm-augmentation of yang and qi [13–15]. Based on these principles, syndrome-based CHM treatments given to FAPS patients generally include (i) warming the interior organ and dispelling cold; (ii) unblocking the liver, reliving stagnation, and regulating qi movement; (iii) cooling heat, dry dampness, relieving stagnation; (iv) warming the stomach and intestine, augmenting qi, and strengthening the spleen to stop pain; (v) nourishing the vital energy of spleen qi [16–20].

In this review article, we mainly presented a Chinese medical view about the etiology and therapy of FAPS, investigate the clinical study on CM for FAPS to reveal the single herbs, Chinese medicine formula, internal application of CM, and so forth, and tried to discuss the scientific basis of CM so as to provide a better understanding of CM for FAPS.

## 2. Material and Methods

Many CHM interventions have been applied for the treatment of FAPS. However, these reports of the clinical study lacked the benefits of individual interventions or individual types of interventions. Another problem is that since the field of FAPS is still not emphasized by both scientists and clinicians, the research in FAPS has very low quality. There is limited evidence-based information from studies specifically designed for the treatment of FAPS. In order to investigate the whole picture of CHM applications in the treatment of FAPS based on syndrome-based differentiation, especially the single herbs, Chinese medicine formula, and CM syndrome, we systematically reviewed all the available data from current databases including clinical trials, clinical observational studies, case studies, and case reports. Based on the large data sets of conventional medicine literature (PubMed, Ovid, etc.) and traditional Chinese medicine literature (SinoMed, CNKI, etc.), we also applied data slicing algorithm in text mining [20]. Through all the comprehensive data searching and synthesis, we aim to investigate the current clinical practice situation of CHM for FAPS and also the basic research of CHM for FAPS.

### 2.1. Literatures Search

Primary electronic database search is listed as below: all EBM reviews, including Cochrane DSR, ACP Journal Club, DARE, CCTR, CMR, HTA, and NHSEED (from inception to Dec. 2015); Embase 1980–Dec. 2015; Embase Classic 1947 to Dec. 2015; PubMed (from inception to Dec. 2015); Ovid MEDLINE(R) 1950–Dec. 2015; Ovid OLDMEDLINE(R) 1948–1965; SinoMed (1978–Dec. 2015); China Journals Full-Text Database (1994–Dec. 2015); CBM Disc (1979–Dec. 2015). Secondary hand search included bibliographic references of identified literatures, textbooks, review articles, and meta-analyses. The search strategy in the study included (abdominal pain^*∗*^ OR chronic abdominal pain^*∗*^ OR functional abdominal pain^*∗*^) AND (herb^*∗*^ OR herbal medicine^*∗*^ OR traditional Chinese medicine OR Chinese medicine OR herbal medicine OR complementary medicine OR naturopathy).

All Chinese-to-English translations were deduced primarily from* the World Health Organization (WHO) Evidence-Based Complementary and Alternative Medicine International Standard Terminologies on Traditional Medicine in the Western Pacific Region* [21].

### 2.2. Interventions to Be Included

Studies of CHM interventions including but not limited to all forms of herbal treatment (single herb, classical formulae, new formulae, herb-derived products, and combination products) should be included.

### 2.3. Trials to Be Included

The trials to be included were as follows: (1) quasi or randomized controlled trials; (2) observational clinical studies; (3) case series or case reports; (4) other types of appropriate research methods.

### 2.4. Data to Be Considered

The data to be considered was as follows: (1) study subjects of any age and gender with FAPS; (2) objective measures by laboratory or imaging techniques; (3) measurement from other informants or nursing staff or patients.

### 2.5. Data Extraction

Two authors searched the databases and selected the relevant publications independently. If there were any disagreements about the eligibility of a study, the two authors would check the study against the selection criteria, discuss its eligibility, and come to a further decision. One author extracted the data and the other checked the extracted data. For each study, the following variables were extracted: study design, sample size, mode of recruitment, sampling and diagnostic procedure, inclusion and exclusion criteria, and participants' characteristics including age, gender, and duration. TCM patterns, TCM treatment principles, treatment regimen, and outcome of TCM treatments were obtained.

### 2.6. Quality Assessment

For RCTs of CHM, methodological quality will be assessed using the Jadad scale. The Jadad scale evaluates a study in terms of the description of randomization, blinding, and dropouts. Points are awarded if the study is described as randomized, 1 point; has an appropriate randomization method, 1 point; is described as double-blind, 1 point; uses appropriate blinding method, 1 point; and has description of withdrawals and dropouts, 1 point. The Jadad scale ranges from 1 to 5, and RCTs with a score from 3 to 5 are regarded as better quality trials.

### 2.7. Text Data Mining

 Besides the systematic literature searching, we also conducted text data mining. The text data mining was conducted by filtering the biomedical literature on FAPS in SinoMed (http://www.sinomed.ac.cn/) on Feb. 25, 2016, and we downloaded associated literature data set containing items. We also applied dictionary-based data slicing algorithm which is constructed on the principle of cooccurrence; we filtered the downloaded literature data with traditional Chinese medicine associated keywords, for example, Chinese herbal medicine, Chinese patent medicine, and TCM syndrome which are collected from textbooks and the Internet [20]. This data mining is a good supplement for literature searching since it will provide some insights into the quantitative relationship between the individual herbs and formula in the treatment of FAPS.

## 3. Results and Discussion

### 3.1. Clinical Study and Chinese Medicine Interventions for FAPS

**Figure 1:**
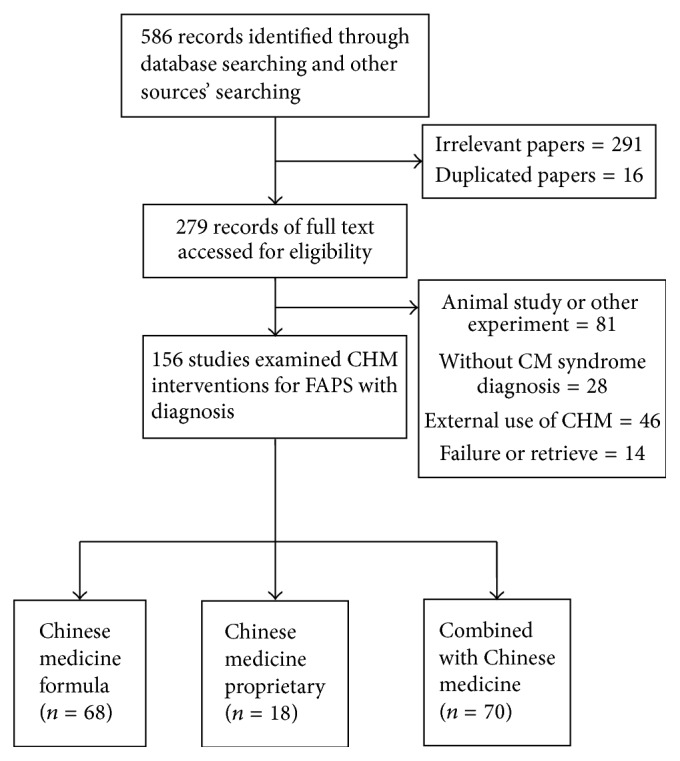
Flowchart of literature selection logistics.

Totally, we accessed 586 records according to our searching criteria, of which 16 were duplicate records and 291 were excluded for reasons of irrelevance. The full text of 279 articles was retrieved for detailed assessment, of which 123 were excluded for various reasons (Figure 1). Of the included 156 studies which fulfilled the inclusion and exclusion criteria, 68 were on Chinese medicine formula, 18 were on Chinese medicine proprietary, and 70 were on a combination of Chinese herbal medicine with conventional treatment. Sample size of the 156 studies ranged from 30 to 210.

### 3.2. TCM Syndrome Category and Treatment Criteria

From the included articles, we totally retrieved 53 different TCM syndrome diagnoses from 156 individual studies. The most common pattern was* liver qi depression*, which was diagnosed in 407 subjects (frequency among all the studies = 37, percentage among the top five diagnoses = 32.5%); it was followed by* liver qi invading the stomach* (subjects = 217, frequency = 24, 21.1%),* liver depression and spleen deficiency *(subjects = 189, frequency = 21, 18.4%),* qi stagnation due to cold *(subjects = 167, frequency = 16, 14.0%), and* spleen-stomach deficiency cold *(subjects = 148, frequency = 14.0%). The results were listed in Table 1.

In all the commonly applied TCM syndromes, we could analyze that, among the top five syndromes, three belonged to excessive syndrome and the other could be categorized as combination of deficiency and sufficiency or deficient syndrome. The result was consistent with other studies by other researchers of syndrome distribution among FAPS patients, especially the elderly and children [17–19].

### 3.3. TCM Single Herbs and TCM Syndrome-Based Chinese Herbal Formulae

The top ten most frequently used herbs and their action were listed in Table 2. The top ten single herbs are Radix Ginseng, Rhizoma Atractylodis Macrocephalae, Pericarpium Citri Reticulatae, Semen Arecae, Lignum Aquilariae Resinatum, Radix Linderae, Rhizoma Corydalis, Radix Aucklandiae, Rhizoma Zingiberis Recens, and Radix Glycyrrhizae. Among 68 studies on Chinese herbal formulae, the most frequently used Chinese herbal formulae based on syndrome diagnosis were Si-Mo-Tang and its modification (number of frequency among all the studies, *N* = 28, percentage among the top five formulae, *p* = 24.6%). It was the same as Tong-Xie-Yao-Fang (*N* = 28, *p* = 24.6%) and followed by Wen-Dan-Tang (*N* = 21, *p* = 18.4%), Xiang-Sha-Liu-Jun-Zi-Tang (*N* = 19, *p* = 16.7%), and Xiao-Yao-San (*N* = 18, *p* = 15.8%). The most five commonly used TCM syndrome-based Chinese herbal formulae and their indications were summarized in Table 3. From this summary, we could clearly notice that the frequency of single herbs is consistent with the combination of the mostly used Chinese herbal formulae. The data mining of Chinese herbal formulae and single herbs was very helpful for us to further discover the mechanisms of TCM in the treatment of FAPS and its potential new combination.

Attention should also be paid to the fact that many studies regarding Chinese herbal formula or Chinese herbal medicine proprietary did not provide the standard criteria or even the complete diagnosis criteria of the syndrome. The results from these studies could not be compared or repeated if the diagnostic criteria varied or were penurious.

### 3.4. Pharmacological Study of Chinese Herbal Medicine on FAPS

Although abundant clinical studies, which has been elaborated as above, have investigated the efficacy of Chinese herbal medicine in treating FAPS, studies on its scientific evidence are not yet available. This may be due to the insufficient understanding of the pathophysiology of FAPS, as well as a scientifically convincing preclinical model being available. Few pathophysiological studies were conducted particularly on clinical FAPS patients, as it is not easy, in a biomedical aspect, to completely discriminate the pathological features of FAPS from those of severe IBS. Indeed, patients with FAPS have a great similarity in symptoms to those with severe IBS. Both FAPS and IBS patients are commonly suffering chronic abdominal pain, which is a multidimensional sensation of sensory, emotional, and cognitive experience. The chronic pain could be explained by the neurophysiological malfunction at the afferent, spinal, and central nerve systems (CNS) [22]. Illustration of the neurophysiological basis of chronic pain in FAPS is scarce; however, data derived from studies on patients with severe IBS revealed that perception of chronic pain comes from a central hypersensitivity and hypervigilance of central turnup of abnormal peripheral input of the gut. The perception of pain can be peripheral or central in alternative scenarios, which reflect either evaluated transmission by gut afferents in response to various stimuli or evoked interpretation of normal transmit accurate information by the CNS [4]. Compared with IBS, with which patients have a certain degree of disorder in peripheral input, FAPS is rendered by predominantly central pathophysiology. A recent study conducted with adults with IBS and FAPS observed that an IBS patient has lowered rectal thresholds in response to rectal balloon distention, while a FAPS patient renders normal perceptual thresholds, indicating the great role of CNS upregulation of incoming afferent signals in perception of chronic pain in FAPS patients [23]. Grover and Drossman summarized the following mechanisms that dominate pathophysiology of chronic pain: (1) ascending visceral pain transmission; (2) peripheral amplification of afferent signals; (3) descending pain modulation; and (4) central amplification and psychiatric factors [2].

Scanty information is available about establishment of an animal model of FAPS, which imposes restriction on the pharmacological study of potential therapy for the disorder. An animal model has been established by slow-release emulsion of morphine (10 mg/kg) for 8 days to develop narcotic bowel-like syndrome [24]. Although it was claimed to be a successful animal model recently [4], this established model does not specifically imitate the pathophysiological condition of FAPS as far as possible [24]. The insufficient development of a preclinical FAPS model largely limits the scientific study of Chinese herbal medicine. Although clinical experiences of ancient and modern Chinese medicine physicians have addressed the effective use of medicinal herbs in treating FAPS, most of preclinical study cannot well discriminate the pharmacological effect of Chinese herbal medicine in pathophysiological context. In this case, we include laboratory tests that were studied on the relief of FAPS-associated symptoms by Chinese herbal medicine. Both pure compounds naturally occurring in medicinal herbs, extracts of single herb and composite herbal formula, were included.

#### 3.4.1. Chinese Herbal Medicine as Antinociceptive with Central Regulation

In a study with classical Chinese medicine formula Tong-Xie-Yao-Fang (TXYF), Hu and colleagues revealed that the formula can significantly relieve experimental visceral hypersensitivity. TXYF significantly decreased serotonin (5-HT) levels in serum and corticotrophin releasing factor (CRF) concentrations in the brain. The pharmacological effect of TXYF is largely dependent on the substance P (SP) expression in the colon mucosa, indicating that the activity of TXYF is associated with central mechanism of brain-gut axis regulation through decreasing the expression of 5-HT and SP in the periphery and that of CRF in the center [25]. Methanol extract of* Kaempferia galangal* (200 mg/kg, p.o.) markedly demonstrated the antinociceptive action to relieve abdominal pain. Both central and peripheral mechanism are involved. The extracts may act as agonist of opioid receptors [26]. Extract of* Hedyotis corymbosa* (Linn.) Lam. (50–200 mg/kg, p.o.) exhibits antinociceptive effect and can relieve abdominal pain in an opioid receptor-dependent manner, which indicates an involvement of central and peripheral mechanism of action [27]. The fractions (ethanol, ethyl acetate, chloroform, and n-hexane) and crude ethyl acetate extract of* Carpolobia lutea* (Polygalaceae) (770 mg/kg, i.p.) can potently relieve abdominal pain in an animal model. Its mechanism of action can be both central and peripheral. The neurogenic (0–5 min) algesia was significantly blocked by the extract, indicating that it can act through opioid receptors which were more centrally than peripherally located. However, reduction on proinflammatory factors by* Carpolobia lutea* treatment reveals its peripheral antinociceptive effect [28].* Senecio rufinervis* essential oil has central and peripheral analgesic effect to relieve abdominal pain. However, the central effect of this essential oil is not as potent as the peripheral one because it does not exhibit any sedative or muscle relaxant property [29]. Carvacrol (5-isopropyl-2-methylphenol), a monoterpenic phenol present in the essential oil of oregano and thyme, exhibited suppression of abdominal pain in an animal model (50–100 mg/kg, p.o.). Mechanistic study revealed that though the pharmacological effect of carvacrol is not mediated by opioid receptor or NO, it can definitely reduce centrally associated intestine neurogenic pain, indicating its antinociceptive effect might involve central regulation [30]. Wang et al. studied the antinociceptive effect of tanshinone IIA on visceral pain induced by chronic pancreatitis (CP). Tanshinone IIA attenuates CP-induced pain via downregulation of spinal high mobility group box 1 (HMGB1) protein and Toll-like receptor 4 (TLR4) expression in the spinal cord, indicating a central regulation underlying its antinociceptive effect [31].

#### 3.4.2. Chinese Herbal Medicine Alleviates Abdominal Pain Majorly by Peripheral Mechanism

Tjong and colleague found that extract of Coptidis Rhizoma can reduce irritable bowel syndrome- (IBS-) associated pain. Coptidis Rhizoma increased pain threshold response and attenuated Electromyogram (EMG) activity via lowering 5-HT release and cholecystokinin (CCK) expression in the colon, which in turn peripherally reduced visceral perception [32].* Lepidium sativum* crude extract (100–300 mg/kg, p.o.) exhibits antispasmodic activities. Study on an isolated rat ileum indicated that this effect could be regional and blockade of muscarinic receptors and calcium channels may be involved [33]. Oral administration of* Cyperus rotundus* extracts (400 mg/kg, p.o.) reduces abdominal pain in mice, which may be associated with a peripheral inhibition of the effect or the release of endogenous substances (arachidonic acid metabolites) that excite pain nerve endings [34]. Friedelin (40 mg/kg, p.o.) isolated from* Azima tetracantha* Lam. can suppress abdominal pain in an animal model. This effect could not be blocked by naloxone, indicating that opioid receptor is not involved and the antinociceptive effect of friedelin may be peripheral [35]. Marrubiin (30.0 *μ*mol/kg, i.p.) isolated from* Marrubium vulgare* has potent antinociceptive effect in relieving abdominal pain. This effect of marrubiin cannot be related to the inhibition of cyclooxygenase products derived from the arachidonic acid pathway or the participation of the opioid system. Analysis using different models revealed that the pharmacological effect of marrubiin may involve peripheral mechanism [36].

#### 3.4.3. Chinese Herbal Medicine That Directly Treats Visceral Pain

A Chinese medicine compound formula, Sunqingwan watered pill (SWP), can reduce abdominal pain caused by ulcerative colitis. The anticolitis effect of SWP may be largely dependent on its protection on the colon through anti-inflammatory mechanisms [37]. Another Chinese medicine formula SWT5 and its component herbs* Angelica* root, chamomile flower, and liquorice root produce antispasmodic activities to relieve upper abdominal pain. The mechanism underlying its effect is independent of the nerve system as fast sodium channel blocker tetrodotoxin, the synaptic transmission blocker *ω*-conotoxin GVIA, or muscarinergic antagonist atropine has minimal block on its pharmacological action. It may involve a direct regulation of smooth muscle cells of the stomach to exert multiple, region-specific effects on gastric motility [38].* Ganoderma lucidum* polysaccharides have potent activity in relieving chronic pancreatitis-induced abdominal pain, which could be associated with reduced production of inflammatory cytokines such as interleukin-1 beta (IL-1*β*) and interferon-gamma (INF-*γ*) [39]. Glycycoumarin, a known component of Radix Glycyrrhizae, exhibits potent antispasmodic activities on the smooth muscle of the mouse jejunum. This pharmacological effect may be associated with intracellular accumulation of cAMP through the inhibition of PDEs, especially isozyme 3 [40]. Isoliquiritigenin, one of the antispasmodic principles of* Glycyrrhiza uralensis* roots, has similar antispasmodic effect on the mouse jejunum, the mechanism of which is yet independent of accumulation of cAMP/cGMP or inhibition of PDEs [41].

#### 3.4.4. Anti-Inflammation-Mediated Relief of Abdominal Pain by Chinese Herbal Medicine

Ethanolic extract from* Pluchea sagittalis *(Lam.) Cabrera (500–700 mg/kg) exhibits antinociceptive effect at inflammatory phase but not neurogenic phase, as demonstrated by the differential pharmacological activities of the extract in acetic acid-induced abdominal pain model and formalin- or glutamate-induced nociception model. The effect of this extract may be associated with its blockade of expression of proinflammatory factors and activation of its downstream pathways [42]. Extract of* Patrinia villosa* (50–100 mg/kg, p.o.) showed inhibition of acetic acid-induced abdominal pain in mice, which could be associated with reduced liberation of inflammatory cytokines including interleukin-6 (IL-6), interleukin-8 (IL-8), and tumour necrosis factor-alpha (TNF-*α*) [43]. Hydroalcoholic extract (1000 mg/kg) of* Neurolaena lobata* (L.) R. Br. and its chloroform- and hexane-partitioned fractions (100 mg kg/kg) can relieve abdominal pain. This effect is not similar to the action of morphine, indicating a mechanism independent of central or peripheral regulation may be involved. It has been postulated that inhibition of inflammatory factors, such as 5-HT, PEG, and histamine, may mediate its pharmacological effect [44]. Crude extract, fractions, and compounds isolated from* Piper tuberculatum* (3–300 mg/kg p.o.) have antinociceptive effect in reducing acetic acid-induced abdominal constriction. This pharmacological action could be due to inhibition of the release of TNF-*α*, IL-1*β*, and IL-8 by resident peritoneal cells [45]. Triglycerides (TFC) of the fermented mushroom of* Coprinus comatus* (10–30 mg/kg, p.o.) have potent analgesic activity on abdominal pain. This is not related to noninflammatory, central perception of pain but may be dependent on the peripheral reduction of proinflammatory cytokines such as TNF-*α*, IL-1*β*, VEGF-*α*, and IL-17 [46].

## 4. Conclusion

The investigation of CHM treatment for FAPS patients was very limited due to the lack of the clinical studies and also the situation is the same as the basic research so far. In order to describe the enough information in this disorder, we retrieved the data from different designs of clinical studies, ranging from case reports and cohort studies to quasi or randomized controlled trials; the quality of these studies was various and we could not make quantitative comparison. Despite the limitations, our study for the first time summarized the important and practicable data concerning the whole picture of CHM applications in the treatment of FAPS. More high quality studies both clinical and basic research concerning the integration of CM syndrome differentiation and disease with standard and repeatable treatment procedure should be further conducted.

## Figures and Tables

**Table 1:** Top five most commonly used TCM syndromes for FAPS.

TCM syndrome	Therapeutic principle	Number of subjects diagnosed with the diagnosis	Number of frequency among all the studies	Percentage among the total syndrome (53)/top 5 syndrome diagnoses
Liver qi depression *肝气郁结*	Soothe the liver and regulate qi	407	37	69.5%/32.5%

Liver qi invading the stomach *肝胃不和*	Harmonize the liver and stomach	217	24	45.3%/21.1%

Liver depression and spleen deficiency *肝郁脾虚*	Soothe the liver and fortify the spleen	189	21	39.6%/18.4%

Qi stagnation due to cold congealing *寒凝气滞*	Dissipate cold and move qi	167	16	30.2%/14.0%

Spleen-stomach deficiency cold *脾胃虚寒*	Warm the middle and dissipate cold	148	16	30.2%/14.0%

**Table 2:** Action and indication of the ten most frequently used herbs for FAPS.

Chinese name in pinyin	Latin name	Frequency of usage	Action	Indication
Ren Shen	Radix Ginseng	78	Replenish the primordial qi; tonify the spleen and lung; promote fluid production and induce tranquilization	(1) Prostration syndrome of primordial qi (2) Lung qi deficiency syndrome (3) Spleen qi deficiency syndrome (4) Thirst due to qi deficiency and consumption of fluid in febrile disease (5) Palpations, fearful throbbing, insomnia, and dream-disturbed sleep

Bai Zhu	Rhizoma Atractylodis Macrocephalae	53	Invigorate spleen and replenish qi; dry dampness and induce diuresis; stop sweating; prevent abortion	(1) Spleen qi deficiency syndrome (2) Edema, phlegm-fluid retention (3) Spontaneous sweating due to qi deficiency (4) Threatened abortion due to spleen deficiency

Chen Pi	Pericarpium Citri Reticulatae	43	Regulate qi and invigorate spleen; dry dampness and resolve phlegm	(1) Qi stagnation of spleen and stomach (2) Retention of dampness and cough with profuse sputum

Bing Lang	Semen Arecae	41	Expel worms and remove food stagnation; move qi; induce diuresis	(1) Intestinal parasitic disease (2) Dyspepsia and qi stagnation manifested as dysentery with tenesmus (3) Edema and beriberi with the manifestations of swelling and pain

Chen Xiang	Lignum Aquilariae Resinatum	29	Promote qi flow to relieve pain; warm the middle energizer to stop vomiting; warm kidney to improve inspiration	(1) Distending pain in the chest and abdomen (2) Stomach cold causing vomiting (3) Dyspnea of deficiency type

Wu Yao	Radix Linderae	26	Promote qi flow to relieve pain; warm kidney to disperse cold	(1) Chest and abdomen pain syndromes (2) Frequent urination and enuresis

Yan Hu Suo	Rhizoma Corydalis	26	Activate blood; move qi; relieve pain	Stagnation of qi and blood stasis causing pain

Mu Xiang	Radix Aucklandiae	23	Promote qi flow to stop pain; regulate the middle energizer	(1) Spleen and stomach qi stagnation syndromes (2) Large intestine qi stagnation syndrome (3) Liver and gallbladder qi stagnation

Sheng Jiang	Rhizoma Zingiberis Recens	19	Dispel cold, release superficies; warm the middle; arrest vomiting; resolve phlegm and stop cough	(1) Exterior contraction of wind-cold (2) Stomach cold and vomiting (3) Lung cold and cough

Gan Cao	Radix Glycyrrhizae	19	Tonify spleen and replenish qi; dispel phlegm and arrest cough; relieve spasm and pain; clear heat and relieve toxicity; harmonize all medicinals	(1) Spleen qi deficiency syndrome (2) Heart qi insufficient syndrome (3) Cough and dyspnea (4) Spasm in the abdomen and extremities (5) Heat-toxin with ulcers and sore throat; medical or food poisoning (6) Moderating the properties of medicinals

**Table 3:** Summary of the top five most frequently used Chinese herbal formulae for FAPS based on syndrome diagnosis.

English name in pinyin	Composition in pinyin	TCM syndrome	Number of frequency among all the studies	Actions in Chinese medicine
Si-Mo-Tang	Ren Shen Bin Lang Chen Xiang Wu Yao	Liver qi depression	28	Activate qi; lower adverse qi; ease the chest and disperse stagnation

Tong-Xie-Yao-Fang	Bai Zhu Bai Shao Chen Pi Fang Feng	Liver depression and spleen deficiency	28	Reinforce the spleen; reduce the liver; relieve pain and stop diarrhea

Wen-Dan-Tang	Ban Xia Ju Hong Fu Ling Gan Cao Sheng Jiang Zhu Ru Zhi Shi Da Zao		21	Regulate qi; remove phlegm; clear gallbladder heat and harmonize the stomach

Xiang-Sha-Liu-Jun-Zi-Tang	Ren Shen Bai Zhu Fu Lin Ban Xia Chen Pi Mu Xiang Sha Ren Gan Cao		19	Replenish qi to invigorate the spleen; activate qi and eliminate phlegm

Xiao-Yao-San	Chai Hu Dang Gui Bai Shao Bai Zhu Fu Ling Gan Cao Bo He Sheng Jiang	Liver depression and spleen deficiency	18	Soothe the liver to relieve depression; invigorate the spleen and nourish blood
